# Application of peripheral blood routine parameters in the diagnosis of influenza and *Mycoplasma pneumoniae*

**DOI:** 10.1186/s12985-024-02429-4

**Published:** 2024-07-23

**Authors:** Jingrou Chen, Yang Wang, Mengzhi Hong, Jiahao Wu, Zongjun Zhang, Runzhao Li, Tangdan Ding, Hongxu Xu, Xiaoli Zhang, Peisong Chen

**Affiliations:** 1 Department of Laboratory Medicine, The First Affiliated Hospital, Sun Yat-sen University, Guangzhou, 510080 China; 2 Department of Laboratory Medicine, Nansha Division, The First Affiliated Hospital, Sun Yat-sen University, Guangzhou, 511466 China; 3 Department of Laboratory Medicine, Guangdong Province Prevention and Treatment Center for Occupational Diseases, Guangzhou, 510300 China; 4 Department of Pediatrics, The First Affiliated Hospital, Sun Yat-sen University, Guangzhou, 510080 China

**Keywords:** Peripheral blood routine parameters, Influenza, *Mycoplasma pneumonia*, Area under the curve, AUC, Random forest, IV@MP algorithm

## Abstract

**Objectives:**

Influenza and *Mycoplasma pneumoniae* infections often present concurrent and overlapping symptoms in clinical manifestations, making it crucial to accurately differentiate between the two in clinical practice. Therefore, this study aims to explore the potential of using peripheral blood routine parameters to effectively distinguish between influenza and *Mycoplasma pneumoniae* infections.

**Methods:**

This study selected 209 influenza patients (IV group) and 214 *Mycoplasma pneumoniae* patients (MP group) from September 2023 to January 2024 at Nansha Division, the First Affiliated Hospital of Sun Yat-sen University. We conducted a routine blood-related index test on all research subjects to develop a diagnostic model. For normally distributed parameters, we used the T-test, and for non-normally distributed parameters, we used the Wilcoxon test.

**Results:**

Based on an area under the curve (AUC) threshold of ≥ 0.7, we selected indices such as Lym# (lymphocyte count), Eos# (eosinophil percentage), Mon% (monocyte percentage), PLT (platelet count), HFC# (high fluorescent cell count), and PLR (platelet to lymphocyte ratio) to construct the model. Based on these indicators, we constructed a diagnostic algorithm named IV@MP using the random forest method.

**Conclusions:**

The diagnostic algorithm demonstrated excellent diagnostic performance and was validated in a new population, with an AUC of 0.845. In addition, we developed a web tool to facilitate the diagnosis of influenza and *Mycoplasma pneumoniae* infections. The results of this study provide an effective tool for clinical practice, enabling physicians to accurately diagnose and differentiate between influenza and *Mycoplasma pneumoniae* infection, thereby offering patients more precise treatment plans.

**Supplementary Information:**

The online version contains supplementary material available at 10.1186/s12985-024-02429-4.

## Introduction

Acute respiratory infections (ARI) are a significant and widespread cause of morbidity and mortality from infectious diseases, both in my country and globally [1]. These infections are particularly prevalent among preschool and school-age children [2]. Recent reports have highlighted a concerning trend, indicating a notable increase in *Mycoplasma pneumoniae* infections among children under the age of 12 over the past three years [3].

Starting from September 2023, there has been a sudden surge in cases of upper respiratory tract infections among children in China [4–6] and worldwide [7], with a particular impact on children under the age of 5 [8,9]. These infections have the potential to progress into pneumonia, a severe and potentially life-threatening condition. On November 22, 2023, the World Health Organization (WHO) reported a series of pneumonia cases in children’s hospitals in Beijing, Liaoning, and other regions [10]. Further investigation revealed that the primary pathogens responsible for these pneumonia cases were Mycoplasma [11] and influenza viruses [5].

The incidence of *Mycoplasma pneumoniae* and influenza infections typically peaks during the autumn and winter seasons due to their seasonal characteristics [12–14]. Both diseases pose significant health risks to individuals and society [15, 16]. They share common symptoms such as cough, fever, sore throat, and runny nose. In severe cases, they can cause respiratory distress and even lead to death. The overlapping clinical manifestations of *Mycoplasma pneumoniae* and influenza infections pose a diagnostic challenge, especially during seasonal transitions when cross-infection between the two diseases is common and difficult to control [17, 18].

The current diagnostic methods for *Mycoplasma pneumoniae* and influenza, including culture, serological, and molecular methods, have limitations [19, 20]. Culture methods, although feasible in specialized reference laboratories, are time-consuming and unsuitable for making rapid treatment decisions [21]. Additionally, obtaining throat swab samples from children can be challenging, leading to missed detections. Moreover, the high cost of these diagnostic methods presents a barrier, particularly for patients in resource-limited areas.

Given these challenges, there is an urgent need to develop more effective, affordable, and rapid diagnostic methods to improve the current diagnostic landscape. In this context, it is crucial to identify an accurate and swift method for distinguishing between *Mycoplasma pneumoniae* and influenza. Peripheral blood parameters show promise as a simple and cost-effective diagnostic tool [22, 23]. However, no study to date has reported the effective use of new peripheral blood parameters to differentiate between *Mycoplasma pneumoniae* and influenza. This highlights the necessity for further research in this area.

To address this need, we conducted a hospital-based retrospective cross-sectional study at the Nansha Division, First Affiliated Hospital of Sun Yat-sen University. The study aimed to compare clinical symptoms, imaging studies, and peripheral blood markers between influenza and *Mycoplasma pneumoniae*. We hope that our findings will contribute to a more comprehensive understanding of accurately distinguishing between these two disorders, ultimately leading to improved patient outcomes.

## Materials and methods

### Data collection

We retrospectively collected clinical laboratory data from patients who visited the outpatient department of the Nansha Division, First Affiliated Hospital of Sun Yat-sen University between September 1, 2023, and January 15, 2024. Data was collected from digital medical records: age, sex, and routine blood test results at the onset of the illness, such as white blood cells (WBC), red blood cells (RBC), hemoglobin (HGB), hematocrit (HCT), platelets (PLT), mean corpuscular volume (MCV), mean corpuscular hemoglobin (MCH), mean corpuscular hemoglobin concentration (MCHC), lymphocytes# (Lym#), monocytes# (Mon#), eosinophils# (Eos#), basophils# (Bas#) and so on. These were analyzed using the Mindray BC6800 and Mindray BC7500 from Mindray Corporation, Shenzhen, China.

We employed a respiratory pathogen nucleic acid detection kit (PCR-fluorescent probe method, 61,303,101, Sansure Biotech Inc., China) to test throat swabs for the diagnosis of *Mycoplasma pneumoniae* and influenza patients. A Ct value of less than 40 was considered positive.

### Data preprocessing

In this study, a total of 490 cases were collected. To accurately illustrate the critical role of various routine blood test indicators in the diagnosis of *Mycoplasma pneumoniae* and influenza among pediatric patients, the study meticulously excluded cases with incomplete test data, subjects over 12 years of age, patients with concurrent infections of *Mycoplasma pneumoniae* and influenza, individuals with underlying immune or blood disorders, and patients with co-infections from other respiratory pathogens. After data preprocessing, 423 cases were retained for the experiment, including 209 cases of influenza and 214 cases of *Mycoplasma pneumoniae*.

### Model training and validation

We leveraged the Deepwise & Beckman Coulter DxAI platform, an online statistical tool (accessible at http://dxonline.deepwise.com/), for the development of an algorithm based on machine learning. This Deepwise platform is designed to autonomously select appropriate machine learning models, present analytical data, and create a page for online analysis. We chose to implement a series of progressively sophisticated models. These included Random Forest (RF), Multilayer Perceptron (MP), Gradient Boosting (GB), Support Vector Machine (SVM), and Linear Discriminant Analysis (LDA) [24, 25].

The data was divided into training and validation sets in a 7:3 ratio, achieved through random allocation. This division process was repeated across 100 separate iterations for robustness. To further ensure the reliability and reproducibility of IV@MP, we conducted an additional round of validation using a cross-sectional study. This study was sourced from the same hospital, covering the period from January 16th to January 27th, 2024, and adhered to identical diagnostic protocols. Laboratory test results corresponding to this period were processed through the IV@MP algorithm. We assessed the efficacy of IV@MP using several key metrics, including the area under the receiver-operator characteristic curve (AUC), accuracy, recall rate, F1 score, sensitivity, specificity, as well as positive and negative predictive values.

### Statistical analysis

Data analysis was performed using SPSS 26.0. The normality of data distribution was assessed using the Kolmogorov-Smirnov test. The T-test was used to evaluate between-group differences for blood routine test indicators that followed a normal distribution, while the Wilcoxon test was utilized for indicators that did not follow a normal distribution. Sex differences among groups were assessed using the Chi-square test. Data presentation: Continuous variables were expressed as median (P25, P75), and categorical variables were presented as mean ± standard deviation. The diagnostic effectiveness of the models was evaluated through ROC analysis. The performance of each model was elucidated and compared using metrics such as the area under the curve (AUC), accuracy, F1 score, sensitivity, specificity, positive predictive value, and negative predictive value. The Delong test was employed to compare the AUC values of different models, with a *p-value* < 0.05 considered statistically significant.

The research adhered to the principles outlined in the Declaration of Helsinki. Approval for the study was granted by the hospital’s Ethics Committee ([2023]331). Informed consent was not required because only residual samples were collected and tested.

## Result

### Comparative analysis of the clinical characteristics in the test cross-sectional study

The dataset used for building the model comprised 423 patients, with 209 individuals in the influenza virus infection group and 214 in the *Mycoplasma pneumoniae* infection group. The median age of the patients was 6 years, with 241 (57.0%) males and 182 (43.0%) females. There were no significant differences in age and sex between the two groups (*p* > 0.05). In subsequent data analysis, differences in clinical symptoms between the two groups were also observed. The group with influenza virus infection exhibited more pronounced clinical features of fever, runny nose, sore throat, and headache (*p* < 0.05), while patients with *Mycoplasma pneumoniae* infection had more noticeable symptoms of cough and expectoration (*p* < 0.05). Detailed information regarding the age and sex of the two groups, as well as the clinical characteristics of each group, can be found in Table 1.

**Table 1:** The demographic and clinical characteristics of the Influenza virus group (IV) and the *Mycoplasma pneumoniae* group (MP)

Variable	IV (*N* = 209)	MP (*N* = 214)	Total (*N* = 423)	Methods	*p*
**Sex**				Chi-square test	0.705
F	88 (42.1%)	94 (43.9%)	182 (43.0%)		
M	121 (57.9%)	120 (56.1%)	241 (57.0%)		
**Age**				Wilcoxon test	0.129
Mean ± SD	6.311 ± 2.58	5.911 ± 2.27	6.109 ± 2.433		
**`**		`		Pearson’s Chi-squared test	0.000 **
0	0 (0.0%)	71 (33.2%)	71 (16.8%)		
1	209 (100.0%)	143 (66.8%)	352 (83.2%)		
**Cough**				Pearson’s Chi-squared test	0.000 **
0	33 (15.8%)	11 (5.1%)	44 (10.4%)		
1	176 (84.2%)	203 (94.9%)	379 (89.6%)		
**Expectorate**				Pearson’s Chi-squared test	0.000 **
0	148 (70.8%)	113 (52.8%)	261 (61.7%)		
1	61 (29.2%)	101 (47.2%)	162 (38.3%)		
**Runny nose**				Pearson’s Chi-squared test	0.003 **
0	75 (35.9%)	107 (50.0%)	182 (43.0%)		
1	134 (64.1%)	107 (50.0%)	241 (57.0%)		
**Nasal congestion**				Pearson’s Chi-squared test	0.734
0	140 (67.0%)	140 (65.4%)	280 (66.2%)		
1	69 (33.0%)	74 (34.6%)	143 (33.8%)		
**Asthma**				Pearson’s Chi-squared test	0.487
N-Miss	0	1	1		
0	209 (100.0%)	211 (99.1%)	420 (99.5%)		
1	0 (0.0%)	2 (0.9%)	2 (0.5%)		
**Sore throat**				Pearson’s Chi-squared test	0.000 **
0	170 (81.3%)	199 (93.0%)	369 (87.2%)		
1	39 (18.7%)	15 (7.0%)	54 (12.8%)		
**Headache**				Pearson’s Chi-squared test	0.000 **
0	160 (76.6%)	199 (93.0%)	359 (84.9%)		
1	49 (23.4%)	15 (7.0%)	64 (15.1%)		
**otalgia**				Pearson’s Chi-squared test	1.000
N-Miss	1	0	1		
0	208 (100.0%)	213 (99.5%)	421 (99.8%)		
1	0 (0.0%)	1 (0.5%)	1 (0.2%)		

* *p* < 0.05, ** *p* < 0.01

### Comparison of laboratory data differences in the test cross-sectional study

Normality tests were conducted on the laboratory data. The results indicated non-normal distribution for all test parameters, except for Mon#, Neu%, Lym%, RBC, HGB, HCT, MCV, MCH, RDW-CV, MPV, PDW, P-LCR, and PDW-SD. Subsequently, for the test parameters that conformed to a normal distribution, we employed the T-test to compare the laboratory test results between the two groups. For those parameters that did not follow a normal distribution, we utilized the Wilcoxon test for comparison. The detailed results of these comparisons for both sets of test parameters are presented in Table 2 and supplementary Table 1.

**Table 2:** Routine blood test results of the Influenza virus group (IV) and *Mycoplasma pneumoniae* group (MP)

Variable	IV(*n* = 209)	MP(*n* = 214)	*p*	AUC
Lym#	1.050(0.770–1.670)	2.155(1.595–2.833)	0.000 **	0.804
Eos#	0.010(0.000-0.030)	0.120(0.040–0.278)	0.000 **	0.824
Bas#	0.010(0.000-0.010)	0.010(0.010–0.020)	0.000 **	0.721
Mon%	0.098(0.079–0.121)	0.074(0.058–0.088)	0.000 **	0.743
Eos%	0.001(0.000-0.004)	0.014(0.004–0.035)	0.000 **	0.796
PLT	234.000(195.000-283.000)	308.000(245.000-377.000)	0.000 **	0.741
PCT	0.200(0.167–0.237)	0.257(0.209–0.303)	0.000 **	0.732
HFC#	0.030(0.020–0.040)	0.070(0.040–0.110)	0.000 **	0.77
PLT-I	234.000(195.000-283.000)	308.000(245.000-377.000)	0.000 **	0.74
PLR	207.890(143.450–306.100)	145.985(104.515-184.162)	0.000 **	0.708

* *p* < 0.05, ** *p* < 0.01

Lym#: Lymphocyte count; Eos#: Eosinophil count; Bas#: Basophil count; Mon%: Monocyte percentage; Eos%: Eosinophil percentage; PLT: Platelet count; PCT: Plateletcrit; HFC#: High Fluorescent Cell count; PLT-I: Plateletcrit Index; PLR: Platelet to Lymphocyte Ratio, compares platelet and lymphocyte numbers

As per the data presented in Table 2 and supplementary Table 1, the majority of test results demonstrate notable variances between the two groups (*p* < 0.05). The individual test’s area under the curve (AUC), indicating their ability to differentiate between *Mycoplasma pneumoniae* infection and influenza infection, ranged from 0.449 to 0.824. According to the criteria [26], AUC ≥ 0.7 indicates acceptable discrimination ability, so we selected indicators with AUC ≥ 0.7 (Table 2). Individuals with *Mycoplasma pneumoniae* infection have a higher Lym#, Eos#, Bas#, Eos%, PLT, PCT, HFC#, and PLT-I compared to those with influenza infection (*p* < 0.0001). Conversely, individuals infected with the influenza virus exhibit a higher Mon% and PLR as opposed to those suffering from *Mycoplasma pneumoniae* infection (*p* < 0.05).

### Development of the IV@MP algorithm

Following the criteria set by Hosmer and Lemeshow, parameters that demonstrate an AUC value for discriminative efficacy less than 0.70 are deemed inadequate for the identification of DNA viruses, M. pneumoniae, and G − organisms. These authors have posited that AUC ranges of 0.70–0.80, 0.80–0.90, and above 0.90 correspond to acceptable, excellent, and exceptional levels of discrimination capacity, respectively [27]. Hence, for the construction of our model, we opted for metrics such as the Number of Lym#, Eos#, Bas#, Mon%, Eos%, PLT, PCT, HFC#, PLT-I, and PLR, (Table 2). After the process of model refinement, a subset of six pivotal indicators was determined.

**Fig. 1:**
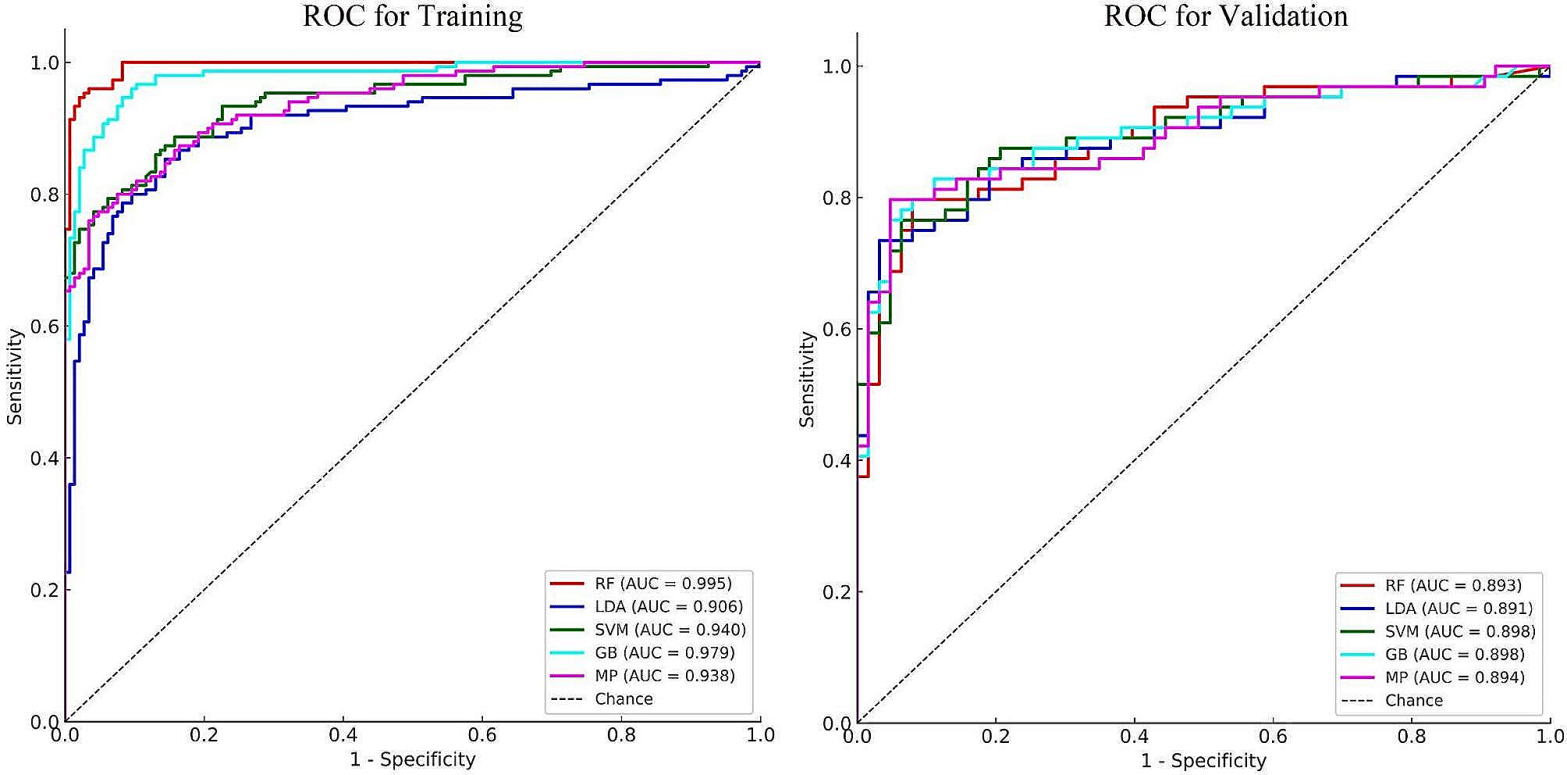
Performance comparisons among five models

To identify the model with the best test efficacy, we constructed five binary classification models based on machine learning (ML). The performance of these five models was evaluated using the DeLong test, and their predictive capabilities are presented in Table 3; Fig. 1.

**Table 3:** Performance Assessment of Five Models in Differential diagnosis prediction for MP and IV

Model		AUC	AC	FI Score	Recall	SE	SP	NPV	PPV
RF	Training	99.48%	96.00%	96.00%	96.00%	96.00%	95.89%	95.89%	96.00%
	Validation	89.35%	80.00%	80.62%	81.25%	81.25%	79.37%	80.65%	80.00%
LDA	Training	90.58%	90.08%	83.99%	78.67%	78.67%	91.10%	80.61%	90.08%
	Validation	89.11%	83.33%	80.65%	78.12%	78.12%	84.13%	79.10%	83.33%
SVM	Training	94.02%	89.71%	85.31%	81.33%	81.33%	90.41%	82.50%	89.71%
	Validation	89.78%	83.08%	83.72%	84.38%	84.38%	82.54%	83.87%	83.08%
GB	Training	97.90%	94.33%	91.41%	88.67%	88.67%	94.52%	89.03%	94.33%
	Validation	89.83%	86.89%	84.80%	82.81%	82.81%	87.30%	83.33%	86.89%
MP	Training	93.83%	88.49%	85.12%	82.00%	82.00%	89.04%	82.80%	88.49%
	Validation	89.41%	84.13%	83.46%	82.81%	82.81%	84.13%	82.81%	84.13%

*In the training data set, the DeLong test showed that the RF model had a larger AUC than the LDA, SVM, GB, and MP models (*p* < 0.01)

*In the validation data set, the AUCs of all the models were not significantly different (*p* > 0.05)

AUC: area under the curve; AC: accuracy; SE: sensitivity; SP: specificity; NPV: negative predictive value; PPV: positive predictive value. RF: Random Forest; LDA: Linear Discriminant Analysis; SVM: Support Vector Machine; GB: Gradient Boosting, MP: Multilayer Perceptron

All five models demonstrated effective predictive performance in distinguishing patients with Mycoplasma pneumonia from those with influenza, as shown in Fig. 1. The results of the DeLong tests for both the training and validation sets of the five models are specifically presented in Table 3. Based on the highest AUC of the RF (Random Forest) model in the training set, and its positive difference compared to the other four models (*p* < 0.01) in Table 4, we ultimately selected the RF model as our predictive algorithm. For convenience, the algorithm based on Random Forest for differential diagnosis of influenza and Mycoplasma pneumonia patients has been named IV@MP.

**Table 4:** Delong test between five models

Training Delong	z	*p*	Validation Delong	z	*p*
RF-LDA	4.984	0.000 **	RF-LDA	0.122	0.903
RF-SVM	4.450	0.000 **	RF-SVM	-0.331	0.741
RF-GB	2.877	0.004 **	RF-GB	-0.498	0.619
RF-MP	4.869	0.000 **	RF-MP	-0.046	0.964
LDA-SVM	-2.732	0.006 **	LDA-SVM	-0.402	0.688
LDA-GB	-4.528	0.000 **	LDA-GB	-0.430	0.667
LDA-MP	-3.091	0.002 **	LDA-MP	-0.207	0.836
SVM-GB	-4.146	0.000 **	SVM-GB	-0.049	0.961
SVM-MP	0.313	0.754	SVM-MP	0.380	0.704
GB-MP	4.576	0.000 **	GB-MP	0.450	0.653

* *p* < 0.05, ** *p* < 0.01

RF-LDA: Delong test between Random Forest and Linear Discriminant Analysis, RF-SVM: Delong test between Random Forest and Support Vector Machine, RF-GB: Delong test between Random Forest and Gradient Boosting, RF-MP: Delong test between Fandom Forest and Multilayer Perceptron, LDA-SVM: Delong test between Linear Discriminant Analysis and Support Vector Machine, LDA-GB: Delong test between Linear Discriminant Analysis and Gradient Boosting, LDA-MP: Delong test between Linear Discriminant Analysis and Multilayer Perceptron, SVM-GB: Delong test between Support Vector Machine and Gradient Boosting, SVM-MP: Delong test between Support Vector Machine and Multilayer Perceptron, GB-MP: Delong test between Gradient Boosting and Multilayer Perceptron

**Fig. 2:**
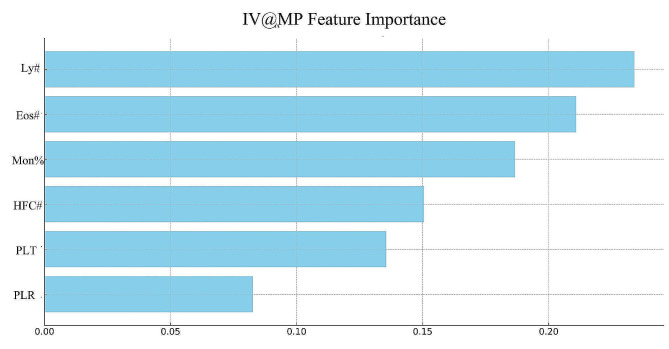
Feature importance in IV@MP

Figure 2 illustrates the significance values of the selected laboratory tests in the IV@MP algorithm. Within this algorithm, six tests were retained. Each of these six tests exhibited an importance greater than 0.3, with Lymphocyte counts (Lym#) having the utmost weight. This was followed, in order of significance, by eosinophil count (Eos#), Monocyte percentage (Mon%), High Fluorescent Cell count (HFC#), platelet count (PLT), and Platelet to Lymphocyte Ratio (PLR).

### Interlaboratory validation

We randomly selected clinical data from patients diagnosed with influenza or mycoplasma pneumonia at the First Affiliated Hospital of Sun Yat-sen University during the same period for IV@MP validation. The characteristics of this validation group are shown in Table 5.

**Table 5:** Characteristics of 106 patients for interlaboratory validation

Variable	ALU(*n* = 58)	MP(*n* = 236)	Total (*N* = 294)	Methods	*p*
**Sex**				Chi-square test	0.817
F	29 (50.0%)	114 (48.3%)	143 (48.6%)		
M	29 (50.0%)	122 (51.7%)	151 (51.4%)		
**Age**				T-test	0.833
Median (0.25–0.75)	6.862 ± 2.228	6.797 ± 2.090	6.81 ± 2.114		

**Fig. 3:**
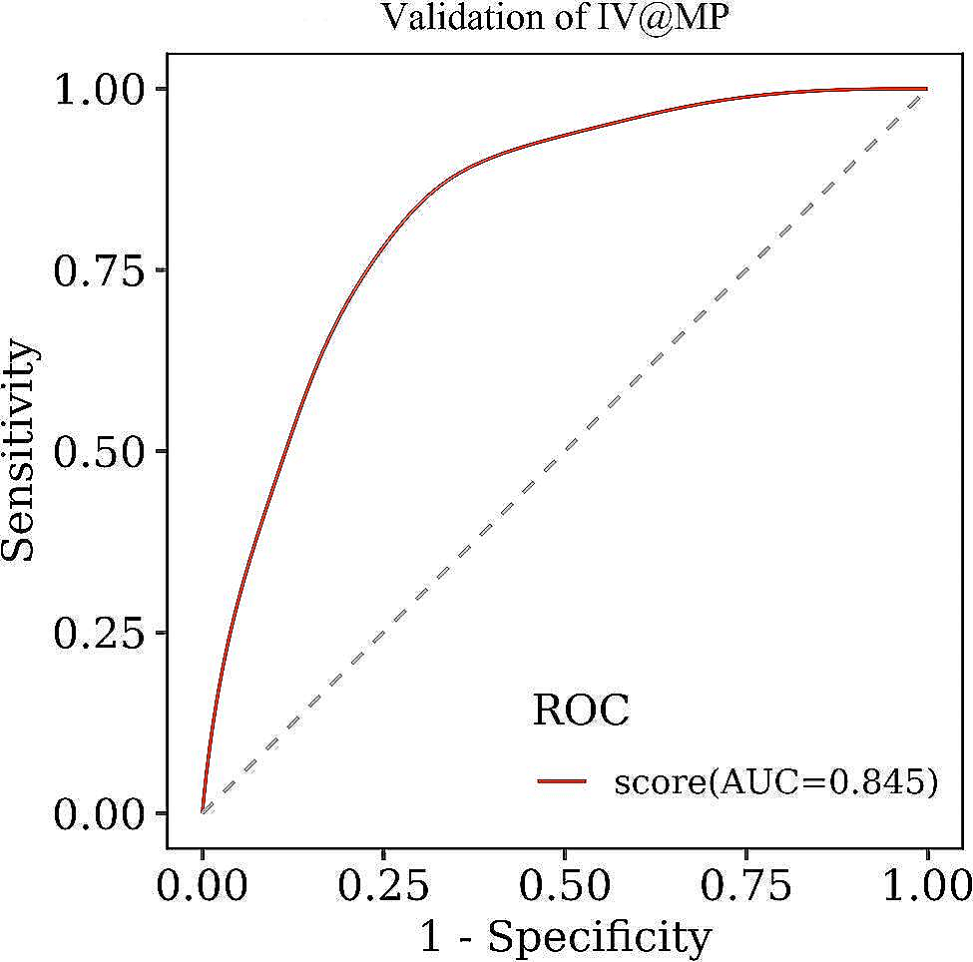
ROC for interlaboratory validation of IV@MP

The diagnostic efficacy of IV@MP in this validation group was summarized in Table 6, with an AUC of 0.845, which is displayed in Fig. 3.

**Table 6:** Interlaboratory validation of IV@MP

	AUC	AC	FI Score	Recall	SE	SP	NPV	PPV
**Interlaboratory validation**	0.845	0.8401	0.8980	0.8771	0.8771	0.6897	0.5797	0.9200

### A webpage tool of IV@MP

**Fig. 4:**
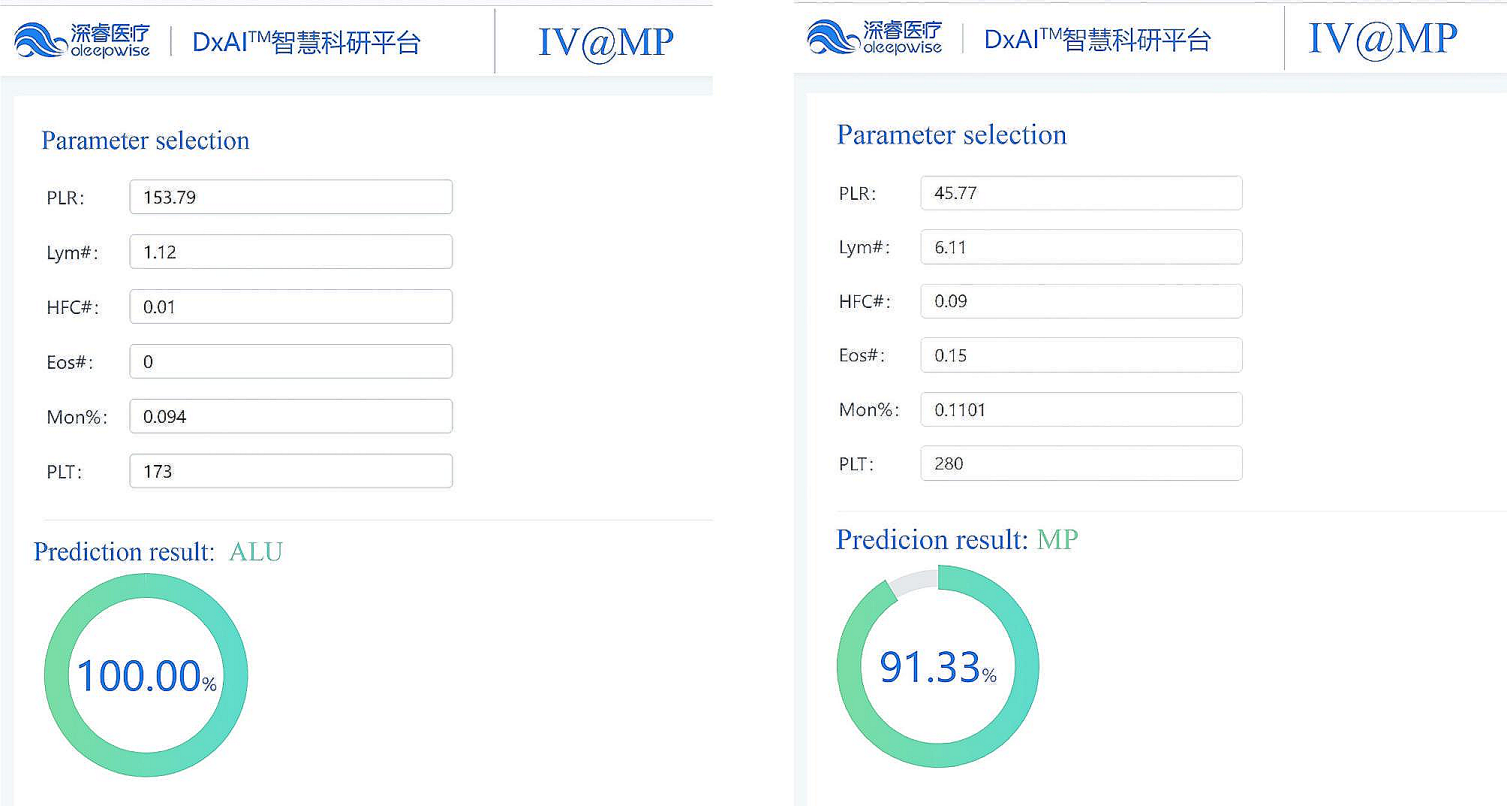
The application of IV@MP

A webpage tool of IV@MP was established (https://dxonline.deepwise.com/prediction/index.html?baseUrl=%2Fapi%2 F&id=42468&topicName=undefined&from=share&platformType=wisdom). A screenshot of the webpage was shown in Fig. 4. Upon inputting the required parameters, the system can determine the likelihood of a patient having either an influenza infection or *Mycoplasma pneumoniae*.

## Discussion

Respiratory infections are one of the major infectious threats faced by the global child population [28, 29]. In developing countries, acute respiratory infections are the leading cause of death among children [30, 31]. Influenza virus and *Mycoplasma pneumoniae* infections have caused multiple pandemics worldwide [32], with each pandemic potentially presenting different symptoms, signs, and laboratory test results. When the influenza virus and *Mycoplasma pneumoniae* co-infect and cause a pandemic, there are often situations of uncertainty.

In this retrospective study, based on medical records, we investigated children aged ≤ 12 years with *Mycoplasma pneumoniae* or influenza virus infection. RT-PCR detection revealed 214 cases of *Mycoplasma pneumoniae* infection, 180 cases of influenza A, and 29 cases of influenza B. Due to the small number of influenza B cases, the influenza A and influenza B cases were combined for statistical analysis. Therefore, the data were divided into two groups: 214 cases in the MP group and 209 cases in the IV group. By comparing the routine peripheral blood parameters of the two, we explored the differences between the two infections to better understand their characteristics and clinical manifestations. This study helps doctors diagnose and treat pediatric respiratory infections more accurately and provides a basis for formulating prevention and control strategies.

Our study first compared the clinical features of pediatric influenza patients and *Mycoplasma pneumoniae* patients. The results showed that the most common symptoms in influenza patients included fever, runny nose, sore throat, and headache, while *Mycoplasma pneumoniae* patients mainly presented with cough, sputum, and wheezing. This finding is consistent with the research results of Wang M [33]. In addition, we found that there was no significant difference in sex distribution among the patient population, whether it was influenza or *Mycoplasma pneumoniae*, which is consistent with the research results of Zhang J [34].

We then analyzed the peripheral blood parameters of influenza and *Mycoplasma pneumoniae* patients and identified parameters with significant differences and AUC ≥ 0.7, including Lym#, Eos#, Bas#, Mon%, Eos%, PLT, PCT, HFC#, PLT-I, and PLR. Although our analysis identified several indicators with relatively high AUC, a single indicator as a standard for disease diagnosis has not yet achieved the expected results. In our expectations, using artificial intelligence models based on big data to perform multifactorial joint diagnostic differentiation between *Mycoplasma pneumoniae* infection and influenza infection can further enhance the efficacy of diagnostic discrimination, making it more effective and practical in clinical applications. There are many interference factors, and the highest AUC is only 0.824. To further explore the role of multiparametric joint diagnostic differentiation between *Mycoplasma pneumoniae* infection and influenza infection, we applied ten effective parameters with AUC ≥ 0.7 to various artificial intelligence models. Ultimately, we found that the combined diagnosis using multiple parameters significantly enhanced the AUC. We then selected the top five models, which showed consistent and superior performance in both training and test sets, namely RF, LDA, SVM, GB, and MP, for the Delong test analysis. These five models have shown effective predictive performance in distinguishing between *Mycoplasma pneumoniae* and influenza patients (AUC > 0.89). However, since the RF model had the highest AUC in the training set and its difference was statistically significant (*p* < 0.01) compared to the other four models, we finally chose the RF model as the prediction algorithm (AUC > 0.99). The random forest model is one type of machine-learning algorithm and is widely used in the biomedical field [35, 36]. Through the refinement process of the random forest model, we identified a subset of six key indicators, including Lym#, Eos#, Mon%, PLT, HFC#, and PLR.

Lymphocytes (Lym) are widely considered to be an important indicator of viral infection [37]. After influenza virus infection, lymphocytes usually decrease, which is consistent with our research results. Compared with *Mycoplasma pneumoniae* patients, influenza patients usually have a decrease in Lym# [2.155(1.595–2.833) vs. 1.050(0.770–1.670), *p* < 0.0001].

Eosinophils (Eos) are considered potential biomarkers for respiratory viral infections [38] and play a role in various important biological processes such as immune regulation [39], autoimmunity [40], and host defense against bacterial and viral infections [41]0.1 Under the action of cytokines, eosinophils in the blood and bone marrow can be recruited to the site of inflammation, thereby producing a large number of immune regulatory factors and pro-inflammatory factors. We found that there were significant differences in eosinophils in influenza and *Mycoplasma pneumoniae* infections. Compared with influenza, *Mycoplasma pneumoniae* patients had higher Eos# [0.010(0.000-0.030) vs. 0.120(0.040–0.278), *p* < 0.0001], which is similar to the report by Yan Q [42].

Monocytes (Mon) play an important role in antiviral immunity. They can directly phagocytose pathogenic microorganisms and can also participate in antiviral immunity through various antibody receptors and lymphokine receptors. When monocytes phagocytose antigens, their carried antigen-determinant clusters can be transferred to T lymphocytes to induce lymphocyte-specific immune responses. When inflammation occurs in the body, it may cause changes in the total number and percentage of monocytes. Therefore, compared with common cold patients, the increase in monocytes in influenza patients is more obvious. Our research also found that the Mon% of influenza patients [0.098(0.079–0.121)] is high, which is similar to the report by Zheng Y [43].

Platelets have many types of surface receptors, which can regulate the interaction between platelets and endothelial cells in an inflammatory state [44]. Some recent reviews have detailed the types and functions of these surface receptors in platelet-mediated responses, especially their interactions with bacteria, bacterial toxins, and endothelial cells [45, 46]. These studies have confirmed the association between platelet count (PLT) and inflammatory response and infection. In our study, we found that the PLT level of *Mycoplasma pneumoniae* patients [308.000(245.000-377.000)] was significantly higher than that of influenza patients [234.000(195.000-283.000)], *p* < 0.0001, indicating that the PLT level may play an important role in distinguishing these two diseases.

High fluorescence intensity cells (HFC) are a new type of peripheral blood parameter. In one study, researchers found that high fluorescence cells (HFC) combined with biochemical immune indicators have application value in identifying the nature of pleural effusion [47] and another study found that the absolute value and percentage of high fluorescence intensity cells have a certain value in the differential diagnosis of benign and malignant pleural effusion [48]. It can reflect the activity of immune cells. In cases of *Mycoplasma pneumoniae*, this activity may be more obvious [0.070(0.040–0.110)], compared with influenza cases, the activity is lower [0.030(0.020–0.040)], *p* < 0.0001. On the other hand, PLR (platelet to lymphocyte ratio) has been widely reported to be associated with respiratory diseases [49, 50] and participates in systemic inflammation and immune responses. Our research found that the PLR level of influenza cases [207.890(143.450–306.100)] was higher than that of *Mycoplasma pneumoniae* cases [145.985(104.515-184.162)], *p* < 0.0001. Therefore, PLR may be a valuable indicator to distinguish these two diseases.

We found that the indicators retained by the model have special significance for differentiating between *Mycoplasma pneumoniae* infection and influenza infection, but the model’s algorithm requires further validation. Consequently, we attempted to find new cases as an external validation set, using this set to analyze and verify the reliability of the model. We randomly selected a group of cases from the First Affiliated Hospital of Sun Yat-sen University, which were tested at the same time with the same equipment. These cases strictly met our inclusion criteria, namely, they were under 12 years old and there was no sex difference. In this way, we successfully obtained an external validation set. The development and validation of the IV@MP algorithm based on random forests showed good diagnostic performance, with an AUC of 0.845 in the external validation set. This highlights the potential of our model in assisting clinicians in accurately diagnosing these respiratory infections This result suggests that using combined indicators for diagnosis can better distinguish between the two, thus it has a higher value.

Our findings not only corroborate the universality and efficacy of our model but also underscore its potential in the broader context of respiratory pathogen diagnostics. Given the diverse spectrum of respiratory pathogens, including but not limited to *Mycoplasma pneumoniae*, influenza viruses, respiratory syncytial virus (RSV) [51], adenoviruses [52], human rhinoviruses [53], and the novel coronavirus (SARS-CoV-2) [54], each with distinct biological traits and clinical presentations, our model’s adaptability is particularly noteworthy. The prevalence of these pathogens fluctuates across different demographics and timeframes, emphasizing the importance of a diagnostic tool that can evolve with emerging data. Should ample data for other pathogens become available, employing a similar methodology could extend our diagnostic reach, thereby amplifying the comprehensiveness and effectiveness of our diagnostic toolkit.

## Conclusion

In summary, our research demonstrates that peripheral blood parameters can aid in the auxiliary diagnosis of influenza and *Mycoplasma pneumoniae*, offering a simplified and cost-effective approach compared to conventional methods. The development of online diagnostic tools for these infections represents a significant step toward improving accuracy and patient management.

### Electronic supplementary material

Below is the link to the electronic supplementary material.

Supplementary Material 1

## Data Availability

No datasets were generated or analysed during the current study.
