# Supplementary material for: Application of peripheral blood routine parameters in the diagnosis of influenza and Mycoplasma pneumoniae
Source: Virol J. 2024 Jul 23;21:162. doi: 10.1186/s12985-024-02429-4 (PMC11267962; doi:10.1186/s12985-024-02429-4)
Supplement: Supplementary file 1 — Supplementary Material 1 [file 12985_2024_2429_MOESM1_ESM.docx]

**Supplementary Table 1 Routine blood test results of the Influenza virus group (IV) and *Mycoplasma pneumoniae* group (MP)**

| **Variable** | **IV(n=209)** | **MP(n=214)** | ***p*** | **AUC** |
| --- | --- | --- | --- | --- |
| WBC | 6.480(4.570-8.800) | 8.375(6.173-11.225) | 0.000 ** | 0.670 |
| Neu# | 4.340(2.950-6.360) | 4.715(3.395-7.452) | 0.010 * | 0.572 |
| Mon# | 0.662±0.287 | 0.661±0.324 | 0.975 | 0.518 |
| IMG# | 0.010(0.000-0.020) | 0.010(0.010-0.020) | 0.001 ** | 0.589 |
| Neu% | 0.685±0.139 | 0.614±0.142 | 0.000 ** | 0.656 |
| Lym% | 0.208±0.124 | 0.285±0.133 | 0.000 ** | 0.689 |
| Bas% | 0.001(0.000-0.002) | 0.002(0.001-0.003) | 0.000 ** | 0.661 |
| IMG% | 0.001(0.001-0.002) | 0.001(0.001-0.002) | 0.220 | 0.533 |
| RBC | 4.595±0.439 | 4.620±0.493 | 0.574 | 0.521 |
| HGB | 126.517±9.642 | 125.383±10.567 | 0.250 | 0.474 |
| HCT | 0.376±0.027 | 0.370±0.029 | 0.039 * | 0.551 |
| MCV | 82.226±6.574 | 80.724±7.707 | 0.032 * | 0.572 |
| MCH | 27.699±2.460 | 27.373±2.969 | 0.220 | 0.533 |
| MCHC | 337.000(334.000-341.000) | 339.000(334.000-344.000) | 0.007 ** | 0.575 |
| RDW-CV | 0.134±0.009 | 0.134±0.011 | 0.913 | 0.531 |
| RDW-SD | 40.000(38.500-41.300) | 39.200(37.625-40.775) | 0.001 ** | 0.597 |
| MPV | 8.612±0.860 | 8.383±0.844 | 0.006 ** | 0.579 |
| PDW | 15.702±0.359 | 15.699±0.377 | 0.926 | 0.492 |
| P-LCC | 37.000(30.000-46.000) | 46.000(35.000-57.750) | 0.000 ** | 0.646 |
| P-LCR | 0.172±0.060 | 0.160±0.056 | 0.035 * | 0.560 |
| HFC% | 0.400(0.200-0.600) | 0.800(0.400-1.400) | 0.000 ** | 0.695 |
| WBC-D | 6.370(4.560-8.530) | 8.345(6.032-11.235) | 0.000 ** | 0.668 |
| WBC-N | 6.480(4.570-8.800) | 8.375(6.173-11.225) | 0.000 ** | 0.670 |
| TNC-D | 6.370(4.560-8.530) | 8.345(6.032-11.240) | 0.000 ** | 0.668 |
| TNC-N | 6.480(4.570-8.800) | 8.375(6.173-11.225) | 0.000 ** | 0.670 |
| NLR | 3.870(2.440-6.470) | 2.315(1.488-3.645) | 0.000 ** | 0.682 |
| Micro# | 0.100(0.050-0.230) | 0.125(0.070-0.300) | 0.012 * | 0.570 |
| Micro% | 2.100(1.200-5.000) | 2.750(1.500-6.400) | 0.012 * | 0.570 |
| Macro# | 0.090(0.070-0.110) | 0.085(0.060-0.100) | 0.100 | 0.546 |

* *p*<0.05, ** *p*<0.01

WBC, White Blood Cell count; Neu#, Neutrophil count; Mon#, Monocyte count; IMG#, Immature Granulocyte count; Neu%, Neutrophil percentage; Lym%, Lymphocyte percentage; Bas%, Basophil percentage; IMG%, Immature Granulocyte percentage; RBC, Red Blood Cell count; HGB, Hemoglobin; HCT, Hematocrit, measures the proportion of blood volume occupied by red blood cells; MCV, Mean Corpuscular Volume; MCH, Mean Corpuscular Hemoglobin; MCHC, Mean Corpuscular Hemoglobin Concentration; RDW-CV, Red Cell Distribution Width - Coefficient of Variation, and RDW-SD, Red Cell Distribution Width - Standard Deviation, both measure the variation in red blood cell size; MPV, Mean Platelet Volume; PDW, Platelet Distribution Width; P-LCC, Platelet Larger Cell Ratio, and P-LCR, Platelet Large Cell Ratio, both calculate larger platelets ratios; and HFC%, High Fluorescent Cell percentage, quantify high fluorescent cells; WBC-D, White Blood Cell - Digital, and WBC-N, White Blood Cell - Nucleated, both represent white blood cell counts; TNC-D, Total Nucleated Cell - Digital, and TNC-N, Total Nucleated Cell - Nucleated, count nucleated cells; NLR, Neutrophil to Lymphocyte Ratio, compares the numbers of neutrophils and lymphocytes; Micro#, Microcytic Cell count, and Micro%, Microcytic Cell percentage, both measure microcytic cells; Macro#, Macrocytic Cell count, and Macro%, Macrocytic Cell percentage, enumerate macrocytic cells; PDW-SD, Platelet Distribution Width - Standard Deviation, indicates the variation in platelet size.
